# Evaluation of the Effect of Adding Various Polymeric Rheological Agents on the Physical and Mechanical Properties of Calcium Silicate Cement

**DOI:** 10.1155/ijod/4002750

**Published:** 2026-09-09

**Authors:** Saeede Zadsirjan, Mojgan Feli, Nazanin Zargar, Golnaz Tayebi, Farhood Najafi, Soolmaz Heidari, Negar Parvaneh Dehkordi, Shokoufeh Borhan

**Affiliations:** ^1^ Iranian Centre for Endodontic Research, Research Institute for Dental Sciences, Shahid Beheshti University of Medical Sciences, Tehran, Iran, sbmu.ac.ir; ^2^ Department of Endodontics, School of Dentistry, Shahid Beheshti University of Medical Sciences, Tehran, Iran, sbmu.ac.ir; ^3^ Department of Dental Biomaterials, School of Dentistry, Tehran University of Medical Sciences, Tehran, Iran, tums.ac.ir; ^4^ Department of Resin and Additives, Institute for Color Science and Technology, Tehran, Iran, irancolorinstitute.com; ^5^ Department of Operative Dentistry, School of Dentistry, Qazvin University of Medical Sciences, Qazvin, Iran, qums.ac.ir; ^6^ School of Dentistry, Shahid Beheshti University of Medical Sciences, Tehran, Iran, sbmu.ac.ir; ^7^ Department of Materials, Chemical and Polymer Engineering, Buein Zahra Technical University, Buein Zahra, Qazvin, Iran, bzte.ac.ir

**Keywords:** calcium silicate, PCE, PVA, PVP

## Abstract

**Objectives:**

The effect of polymer addition on the properties of calcium silicate cement (CSC) was evaluated.

**Materials and Methods:**

The powder component was based on calcium silicate. The liquid component contained distilled water (control group) and distilled water with polyvinyl alcohol (PVA), polyvinylpyrrolidone (PVP), and polycarboxylate ether (PCE) polymers separately or in combination (PVA + PCE, PVP + PCE) at concentrations of 1 and 3 wt%. In addition to the evaluation of handling properties, some analyses were performed, including setting time, compressive strength, flowability, and injectability. The best‐performing groups were selected as the final groups. The specimens were immersed in distilled water and simulated body fluid (SBF) for 1 and 7 days to evaluate the amount of calcium ion release, weight changes, and hydroxyapatite formation. One‐way ANOVA, repeated measures ANOVA, and Tukey tests were used for statistical analysis (α = 0.05).

**Results:**

Based on the handling evaluation and the results of the setting time, compressive strength, flowability, and injectability analyses, the PVA 3% and PVP 3% groups were selected as the optimal groups and subjected to further analysis. The PVA 3% group showed the highest calcium ion release after 1 day of immersion, while no significant difference was observed among the groups on the seventh day. All three groups (PVA 3 wt%, PVP 3 wt%, and control) showed the formation of calcium phosphate deposits after immersion in SBF.

**Conclusion:**

The use of PVA 3% and PVP 3% polymers separately in the liquid component of CSC improved the properties of this cement.

## 1. Introduction

Calcium silicate cements (CSCs) are hydraulic cements that have always been of interest in bone and dental tissue engineering due to their properties, such as biocompatibility and potential for hard tissue regeneration. One of the most well‐known CSCs used in dentistry is mineral trioxide aggregate (MTA), which has been shown to be effective in many restorative and endodontic dental treatments, including direct and indirect pulp capping, perforation closure due to its proven bioactivity properties, apexogenesis, apexification, apicoectomy, etc. The four main components of MTA have traditionally been tricalcium silicate, dicalcium silicate, tricalcium aluminate, and tetracalcium aluminoferrite. In newer formulations marketed as White MTA, the content of iron‐containing compounds has been reduced to minimize the risk of tooth discoloration [1].

The setting mechanism of this cement is the result of a series of simultaneous and successive hydration chemical reactions between the liquid and powder components of the cement. In the hydration phenomenon, the main chemical reaction takes place between tricalcium silicate and dicalcium silicate with water, resulting in the formation of hydrated calcium silicate gel and calcium hydroxide [2]. Calcium hydroxide is the main biological product of the cement hydration reaction. In addition to its excellent biocompatibility, it leads to the formation of hydroxyapatite in the biological environment of the body. Hydroxyapatite forms a direct bond with the hard tissue of the tooth and provides a favorable adhesion [3].

However, MTA has some drawbacks. Its gritty or sandy consistency makes it difficult to handle. Furthermore, the presence of porosity in the final structure and the potential for tooth discoloration are concerns. In some products, the long setting time makes this material unsuitable for single‐visit treatment [1, 4]. Efforts have been made to overcome these limitations while maintaining the desirable properties of MTA [1, 5].

Polymers are among the rheological modifiers. High molecular weight, chain entanglement, and the ability to regulate solvent‐polymer interactions are desirable characteristics of these materials [6]. Polyvinyl alcohol (PVA) is a hydrophilic polymer that can serve as an adhesive and a modifier. The addition of small amounts of this polymer to cement‐based materials can have a significant effect on the properties of cement, including improving workability [7]. Polyvinylpyrrolidone (PVP) is a hydrophilic polymer that is used in the manufacture of pharmaceutical tablets as a binder and also as a viscosity enhancer [8]. Both polymers can also act as dispersants, preventing the accumulation of particles or the settling of particles by separating the particles in the solution [7, 8]. Both PVA and PVP are nontoxic and biodegradable [9]. To date, only a limited number of studies have been conducted on these two polymers in the field of bone tissue engineering and dental procedures [10, 11]. Polycarboxylate ether (PCE) is also a hydrophilic polymer with low toxicity and high chemical stability. This polymer is considered a superplasticizer, a material that is added to concrete to improve its properties, including workability and flowability, without having a negative impact on strength as much as possible [12]. To date, only a limited number of studies have been conducted on the use of this polymer in the formulation of materials used in dentistry and medicine [13]. The selection of PCE as a component of the biological cement was based on the findings of Koch et al. [14]. Due to the limited number of studies and the properties investigated, the present study aimed to evaluate the effects of incorporating these polymers on the properties of CSC.

## 2. Materials and Methods

### 2.1. Powder Synthesis

The sol–gel method was employed to synthesize tricalcium silicate (C_3_S), dicalcium silicate (C_2_S), and tricalcium aluminate (C_3_A), separately. The synthesis of powder was performed according to the method described by Moon et al. [15]. For the synthesis of C_3_S, C_2_S, and C_3_A, 0.3, 0.2, and 0.2 mol of calcium nitrate tetrahydrate were used, respectively. The final sintering processes were performed at 1450°C (4 h), 1250°C (4 h), and 1250°C (4 h) for C_3_S, C_2_S, and C_3_A, respectively. The obtained powders were mixed with a ratio of 50 wt% C_3_S: 20 wt% C_2_S: 10 wt% C_3_A. Bismuth oxide and zirconium oxide were also added to the mixture as radiopacifiers. Finally, the obtained powder was ball‐milled for 2 h and sieved through a 400 mesh sieve.

### 2.2. Liquid Preparation

PVA (MW: 72,000) and PVP (MW: 40,000) polymers, both from Merck KGaA (Darmstadt, Germany), and Melflux4930 F polymer, from BASF (Ludwigshafen, Germany) (PCE) at 1 and 3 wt%, were dissolved separately in distilled water. To prepare a 1 wt% PVA solution, distilled water was added to 0.15 g of PVA until the volume reached 15 mL. The solution was then stirred for 30 min. The other solutions were prepared in the same way. Table 1 describes the groups investigated in this study. The powder component was similar for all groups.

**Table 1:** Description of the study groups.

Distilled water (control)
PCE1%
PCE3%
PVP1%
PVP3%
PVA1%
PVA3%
PCE1% + PVP1%
PCE1% + PVP3%
PCE1% + PVA1%
PCE1% + PVA3%

### 2.3. Cement Preparation

Powder and liquid were mixed on a glass plate using a plastic spatula. A scoop of powder containing 0.14 g of powder was mixed with an appropriate amount of liquid. The best cement consistency was taken into account in selecting the correct amount of liquid. It should be noted that the handling of the cements was evaluated by two blind operators.

### 2.4. Physical and Mechanical Properties

#### 2.4.1. Evaluation of Setting Time

The setting time was evaluated by preparing disk‐shaped specimens (*n* = 10, 10 mm diameter and 2 mm height). The setting time test was performed using a Gilmore apparatus (a needle with a diameter of 1.06 mm and a weight of 453.6 g). Specimens were incubated during the test (37°C and 90% humidity). The Gilmore apparatus was applied at 5‐min intervals. The indenter was gradually lowered and placed on the material for a period of 5 s. This process was repeated until no indentation remained on the surface [16]. Based on the results of the setting time test, the optimal groups were selected for further analysis.

#### 2.4.2. Compressive Strength

Cylindrical specimens (4 mm diameter and 6 mm height) were prepared and kept in an incubator (37°C, 90% humidity) for 7 days. The specimens were dried and then subjected to compressive loading (200 kgf, 0.5 mm/min) until fracture using a Universal Testing Machine (UTM; Zwick/Roell, Ulm, Germany). The compressive strength was measured according to the following formula:

σ=F/A,

where *F* represents the maximum load at failure (N) and *A* represents the cross‐sectional area (mm^2^) [16].

### 2.5. Flowability

Two glass slabs with dimensions of 40 × 40 mm, thickness of 5 mm, and weight of 20 g were used for this test. 0.05 ± 0.005 mL of mixed cement was placed in the center of the slab. 180 s after the start of mixing, the second slab was placed on the cement, and a weight of 100 g was placed on it (120 g in total). 10 min after the start of mixing, the slab and the weight were removed, and the small and large diameters of the disk‐shaped cement were measured with the digital caliper. If the difference between the two diameters was more than 1 mm, the test was repeated; otherwise, the average of the two diameters was calculated. The test was performed three times for each group, and the average was calculated [17]. The selected groups proceeded to the next test.

### 2.6. Injectability

To perform the injection test, a 3 mL syringe with a needle diameter of 1.1 mm (19 gauge) was filled with the cement. Compressive force was applied using a UTM to extrude the cements at a rate of 30 mm/min. The maximum force applied against the displacement of the piston was measured. In addition, the injectability of different cements was calculated using the following equation:

Injectability%= volume of the extruded cement/original volume of the cement×100.

Finally, according to the results of the initial tests, the best groups were selected for further analyses.

### 2.7. Handling Properties

Handling characteristics were qualitatively assessed by two operators during material preparation and placement based on ease of mixing, homogeneity, cohesiveness, and ease of manipulation. No formal scoring system or statistical analysis was applied.

### 2.8. Biological Properties

#### 2.8.1. Ion Release Analysis

Ion release analysis was performed using ICP‐OES (Inductively Coupled Plasma–Optical Emission Spectrometry; Vista‐Pro, Varian, Mulgrave, Australia). To prepare the specimens, polyethylene molds with a diameter of 4 mm and a height of 10 mm were used. In order to standardize the mass of the cement, the molds were weighed by a digital balance before and after filling. Each filled mold was immediately immersed in a container containing distilled water (0.01 g/1 cc). The specimens were kept in an incubator (37°C, 90% relative humidity) for 24 h. After this period, the specimens were carefully removed and placed in an equal amount of new distilled water in another container for 7 days. The solutions obtained from each time period were used to determine the pH and ion release.

#### 2.8.2. pH Evaluation

The pH of the specimens immersed in the distilled water mentioned in the ion release analysis was measured by a pH meter (Jenway 3310, Staffordshire, UK) on day 1 and day 7. The pH of each specimen was measured three times, and the average was reported.

#### 2.8.3. Weight Loss

Specimens were prepared as described for the ion release assay. Specimens were placed in the incubator for a period equal to 70% of the final setting time. Then, the set cements were separated from the molds and weighed. Half of the specimens were placed in distilled water for 1 day and the rest for 7 days (*n* = 10). After each period, the specimens were first air dried and then reweighed. The percentage of weight loss was measured as follows:

Weight loss % =Initial weight −Secondary weight/Initial weight×100.

### 2.9. Biomineralization

X‐ray diffraction (XRD) and scanning electron microscopy/energy dispersive spectroscopy (SEM/EDS) analyses were used to investigate the ability of cements to form hydroxyapatite following immersion in simulated body fluid (SBF) solution. SBF was prepared according to the protocol described by Kokubo and Takadama [18]. The solution contains ionic constituents similar to those of human blood plasma, including Na^+^, K^+^, Mg^2+^, Ca^2+^, Cl^−^, HCO^3−^, HPO_4_^2−^, and SO_4_^2−^ [18]. Disk‐shaped specimens of selected cements (based on physical, mechanical, and rheological tests) with a diameter of 10 mm and a thickness of 2 mm were prepared. After 24 h of setting, they were placed in a SBF solution (0.01 g/mL). The specimens were placed in an incubator (37°C, 90% humidity) for 1 and 7 days. XRD analysis (X′Pert PRO MPD, PANalytical, Almelo, Netherlands) was performed to characterize the crystalline phases with specifications of 40 kV, 40 mA, step size of 0.02°, and a range of 5–120° 2Ѳ. The specimens were evaluated by an electron microscope (ESEM Quanta 200, FEI, Hillsboro, OR, USA) equipped with an EDS chemical analyzer to observe the morphology and trace the formation of hydroxyapatite. The specimens were gold‐coated to improve the resolution.

### 2.10. Statistical Analyses

In order to evaluate the normal distribution of the data and the homogeneity of the variances in the studied groups, the Kolmogorov–Smirnov and the Levene’s test were employed, respectively. When *p* was greater than 0.05, one‐way ANOVA and post‐hoc Tukey tests were used to compare the groups. Otherwise, Welch and Games‐Howell tests were used. Repeated measures ANOVA was used to evaluate the results of the different groups at different time points. Additionally, paired‐samples *t*‐test was utilized for the purpose of intra‐group comparison. The significance level was set at α = 0.05.

## 3. Results

### 3.1. Descriptive Observations of Two Operators on Material Handling

In general, the addition of polymers, especially as a single polymer, improved cement handling. The PVP 3 wt% group had better handling properties than the other polymers.

### 3.2. Physical, Mechanical, and Rheological Properties

#### 3.2.1. Setting Time

Significant statistical differences were observed in the pairwise comparisons, except for the six comparisons shown in Table 2. The control group had a significantly longer setting time than the other groups.

**Table 2:** Pairwise comparison of the setting time.

Group	Setting time (min)
Distilled water	173.70 ± 4.39
PCE1%^a^	103.90 ± 2.96
PCE3%^bc^	113.20 ± 1.93
PVP1%^a^	105.60 ± 0.96
PVP3%^cde^	115.60 ± 1.26
PVA1%^be^	112.30 ± 1.70
PVA3%^d^	118.60 ± 1.26
PCE1% + PVP1%	144 ± 4.11
PCE1% + PVP3%^f^	157.80 ± 3.96
PCE1% + PVA1%^f^	153.60 ± 3.16
PCE1% + PVA3%	167.60 ± 3.92

*Note:* Groups with similar lowercase letters had no significant difference (*p* > 0.05).

### 3.3. Compressive Strength

The 3 wt% groups were selected for compressive strength evaluation (despite the longer setting time compared to the 1 wt% groups). This allowed the effect of these polymers on other properties to be evaluated when used at the maximum concentration. In addition, the exclusion of some of the groups prevented further confusion in the interpretation of the results. Pairwise comparisons showed no significant differences (Table 3). As a result, all six groups proceeded to the next step, and no group was excluded at this time.

**Table 3:** There was no statistically significant difference (*p* > 0.05) in the comparison of compressive strength between different groups.

Group	Compressive strength (MPa)
Distilled water	16.96 ± 3.88
PCE 3%	11.24 ± 1.95
PVP3%	13.08 ± 3.45
PVA3%	16.68 ± 5.32
PCE1% + PVP3%	12.21 ± 2.81
PCE1% + PVA3%	16.89 ± 4.22

### 3.4. Flowability

The control group showed the lowest value. The best result was shown by PCE 1% + PVA 3%. However, there was no significant difference between the groups except for the control group and PCE 3%.

Due to the lower compressive strength (statistically insignificant) and lower flow (statistically significant) of the 3 wt% PCE group compared to other groups, particularly the control group, this group was excluded from further testing (Table 4).

**Table 4:** Pairwise comparison between the flowability of the cements.

Group	Flow (mm)
Distilled water	7.60±1.07^c^
PCE 3%	9.08±0.25^b^
PVP 3%	10.77 ± 0.156^a^
PVA 3%	10.48 ± 0.179^a^
PCE 1% + PVP 3%	10.63 ± 0.14^a^
PCE 1% + PVA 3%	10.81 ± 0.14^a^

*Note:* Non‐similar superscript letters indicate that the two groups had a significant difference (*p* < 0.05).

### 3.5. Injectability

The mean and standard deviation of the injectability values of the groups studied are shown in Table 5. The results showed that there was no difference among the different groups. It should be noted that the percentage of injectability was 100% in all groups. However, the best result was obtained with PCE 1% + PVA 3%.

**Table 5:** Mean ± standard deviation of injectability in different groups.

Group	Injectability (*N*)
Distilled water	254.96 ± 24.01
PVP 3%	221.87 ± 34.94
PVA 3%	248.19 ± 13
PCE 1% + PVP 3%	240.51 ± 12.60
PCE 1% + PVA 3%	210.75 ± 16.43

*Note:* No significant difference was observed (*p* > 0.05).

Table 6 shows the results obtained for the selected groups. Finally, according to the comparison of the properties considered as key parameters, the PVA 3% and PVP 3% groups were used for the analyses related to biomineralization.

**Table 6:** Results obtained for the selected groups.

Group	Setting time (min)	Compressive strength (MPa)	Flow (mm)	Injectability (*N*)
Distilled water	173.70 ± 4.39	16.97 ± 3.88	7.60 ± 1.07	254.96 ± 24.01
PVP 3%	115.6 ± 1.26	13.08 ± 3.45	10.77 ± 0.156	221.87 ± 34.94
PVA 3%	118.60 ± 1.26	16.69 ± 5.32	10.49 ± 0.179	248.19 ± 13
PCE1%+ PVP3%	157.80 ± 3.96	12.21 ± 2.81	10.63 ± 0.14	240.51 ± 12.60
PCE1%+ PVA3%	167.60 ± 3.92	16.89 ± 4.22	10.81 ± 0.14	210.75 ± 16.43

### 3.6. Evaluation of Weight Loss, pH, and Ion Release in Distilled Water

#### 3.6.1. Weight Loss

In all groups, the amount of weight loss increased significantly from day one to seven. The PVA 3% group exhibited significantly less weight loss than the other two groups (distilled water and PVP 3%) on both day 1 and day 7, while the difference in weight loss between the distilled water group and the PVP 3% group was not significant (Table 7).

**Table 7:** Mean ± standard deviation of weight loss.

Group	Weight loss (%) (first day)	Weight loss (%) (seventh day)
Distilled water	5.200 ± 1.38^Aa^	9.29 ± 1.53^Ba^
PVA 3%	3.41 ± 1.30^Ab^	7.36 ± 0.95^Bb^
PVP 3%	6.56 ± 0.40^Aa^	10.33 ± 0.80^Ba^

*Note:* The use of different uppercase superscript letters indicates significant differences in each group between two time periods. In contrast, the use of different lowercase superscript letters demonstrates the difference between groups in each time period (*p* < 0.05).

### 3.7. pH Test

The initial pH of distilled water was 5.33. No statistically significant differences were observed among the groups on day 1. On day 7, the pH level of the PVA 3% group was found to be significantly lower than that of the other two groups (distilled water and PVP 3%). However, the pH difference between these two groups was not found to be statistically significant. A significant decrease in the pH level was observed in all three groups from day 1 to 7 (Table 8).

**Table 8:** Mean ± standard deviation of the pH values.

Group	pH (first day)	pH (seventh day)
Distilled water	10.03 ± 0.48^Aa^	9.94 ± 0.48^Ba^
PVA 3%	10.95 ± 0.14^Aa^	8.78 ± 0.39^Bb^
PVP 3%	10.68 ± 0.11^Aa^	10.61 ± 0.09^Ba^

*Note*: The use of different uppercase superscript letters indicates that there are significant differences between the groups in each time period. In contrast, different lowercase superscript letters indicate the difference between groups in each time period.

### 3.8. Ion Release

#### 3.8.1. Ca

When comparing the groups, the PVA 3% group released significantly more calcium ions than the other two groups (distilled water and PVP 3%) on day 1. On day 7, there was no statistically significant difference between the groups. Statistically significant differences were observed in calcium‐ion release between the first and seventh days in all three groups (Table 9).

**Table 9:** Mean ± standard deviation of Ca‐ion release.

Group	Ca release (mg/L) first day	Ca release (mg/L) seventh day
Distilled water	25.25 ± 3.77^Ab^	18.369 ± 1.46^Ba^
PVA 3%	37.25 ± 5.29^Aa^	20.40 ± 1.50^Ba^
PVP 3%	22.27 ± 0.81^Ab^	19.70 ± 0.39^Ba^

*Note:* Different uppercase letters indicate significant differences in each group between two time periods. Different lowercase letters indicate the difference between groups in each time period.

#### 3.8.2. Si

On day 1, the PVA 3% group showed a significantly greater Si release than the other two groups (distilled water and PVP 3%). PVP 3% showed the lowest Si‐ion release on day 1. There was no significant difference between the groups on day 7. In the within‐group comparison, a statistically significant difference between day 1 and day 7 was observed only in the PVA 3% group (Table 10).

**Table 10:** Mean ± standard deviation of Si ion release.

Group	Si release (mg/L) first day	Si release (mg/L) seventh day
Distilled water	10.77 ± 6.04^Aa^	6.29 ± 1.63^Aa^
PVA 3%	29.92 ± 1.97^Aa^	7.11 ± 1.53^Ba^
PVP 3%	5.60 ± 1.09^Ab^	7.75 ± 0.90^Aa^

*Note:* Different uppercase letters indicate significant differences in each group between two time periods. Different lowercase letters indicate the difference between groups in each time period.

#### 3.8.3. XRD

**Figure 1:**
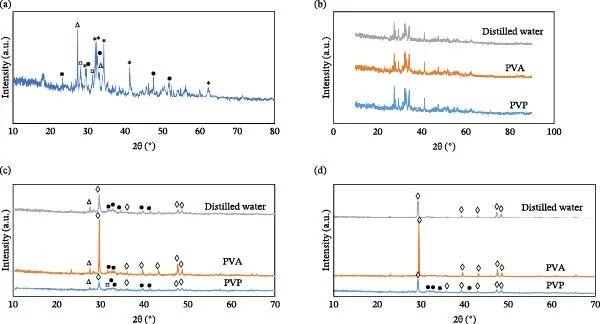
XRD analysis of calcium silicate powder (a); marked crystalline phases: Δ = Bi_2_O_3_, ▫ = ZrO_2_, • = C_2_S,  ^∗^ = C_3_S, ▪ = Ca_2_Al_2_SiO_7_. XRD analysis of the powders following hydration (b). Specimens immersed in SBF for 1 day (c); marked crystalline phases: ◊ = CaCO_3_, • = C_2_S, Δ = Bi_2_O_3_, ▫ = ZrO_2_. Specimens immersed in SBF for 7 days (d); identified crystalline phases: ◊ = CaCO_3_, • = C_2_S, Δ = Bi_2_O_3_, ▫ = ZrO_2_.

The hydrated CSCs and calcium silicate powder showed similar XRD patterns. However, the intensity of the crystalline phases in the cement containing the PVP polymer was higher. The XRD patterns of the surface of the specimens immersed in the SBF solution for 1 and 7 days showed calcium carbonate as the dominant crystalline phase (Figure 1).

#### 3.8.4. SEM/EDS

**Figure 2:**
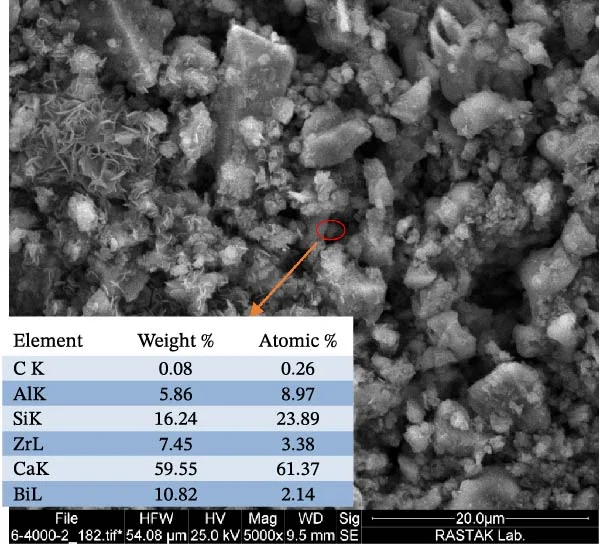
SEM/EDS analysis of the hydrated calcium silicate cement (control group).

**Figure 3:**
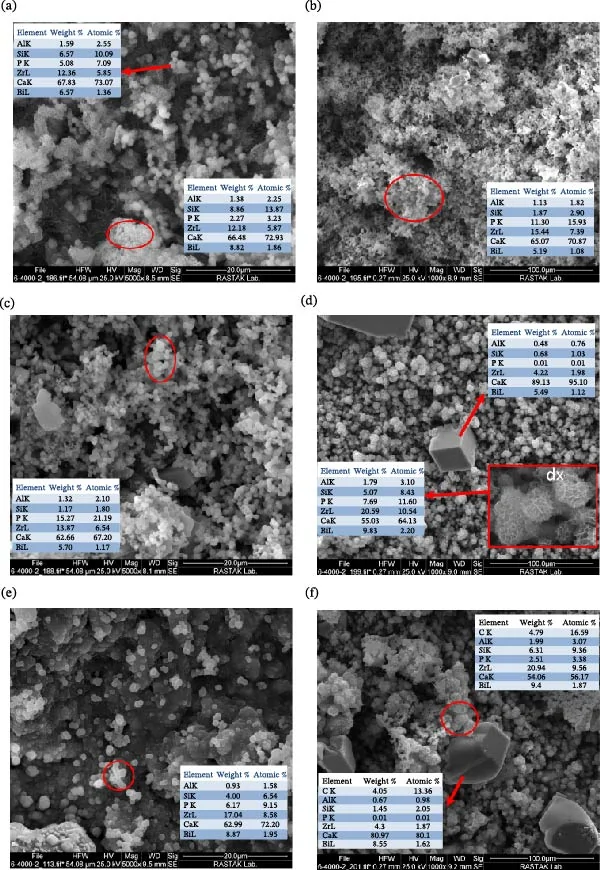
SEM/EDS analysis of the different groups immersed in SBF. (a) Control group after 1 day. (b) Control group after 7 days. (c) PVA 3% after 1 day. (d) PVA 3% after 7 days. (e) PVP 3% after 1 day. (f) PVP3 % after 7 days.

The formation of calcium phosphate deposits with a spherical appearance was confirmed after 1 and 7 days of immersion in SBF. The formation of calcium carbonate deposits with cubic and polyhedral shapes as well as spherulite was observed in different groups (Figures 2 and 3).

## 4. Discussion

In this study, three different types of polymers were used as rheological modifiers in the liquid component to investigate their respective effects on the properties of CSC. The results showed that the addition of each polymer, either individually (up to 3 wt%) or in combination with PCE, improved the consistency and handling properties of the obtained cement. This result is consistent with the findings of Noh et al., who reported that the optimal effect of the PVA polymer on handling occurred between 3 and 5 wt%. However, above 5%, it becomes difficult to mix the powder and liquid homogenously [19]. The PCE polymer used in this study is a comb‐shaped polymer, in which the presence of ether and carboxylate groups leads to the formation of a more effective interaction with cement particles. The use of this plasticizer has been demonstrated to improve handling and workability [13, 20]. It is also used in Biodentine (Biodentine, Septodont, Saint Maur des Fossés, France) [21]. Due to the commercial experience of this material, this polymer was used in combination with two other polymers in this study to investigate the effect of simultaneous use. The results showed that the use of each polymer individually resulted in a more pronounced improvement in cement consistency compared to the simultaneous use with 1 wt% PCE.

In this study, the PCE polymer exhibited the greatest effect in reducing the setting time. It has been postulated that the use of water‐reducing agents, i.e., polymers whose addition leads to a reduction in the amount of water required for the preparation of cement, slows down the hydration process and subsequently increases the setting time of cement [22]. This is in contrast to the findings of the present study. In the current study, the liquid‐to‐powder ratio in the groups containing 3 wt% polymer increased in the following order: distilled water ≈ PVA < PCE ≤ PVP. Different liquid contents were used to obtain the optimum powder‐to‐liquid ratio for each cement formulation and to achieve consistent workability and handling characteristics.

The polymers used have different structural and molecular‐weight characteristics, which may explain the observed differences in their behavior. Despite the slight increase in the liquid‐to‐powder ratio in the PCE and PVP groups and the probable effect on the setting time, these two groups still demonstrated a shorter setting time than the other two groups. Regarding the effect of polymer addition on setting time, conflicting results have been reported in previous studies [13, 19, 23–25]. It is important to note that there are only a few studies that have investigated the effect of polymer addition on the properties of CSCs. Therefore, it is not possible to make an adequate comparison with the results of this study.

It has been demonstrated that the addition of PVA polymer reduces the ratio of liquid to powder in the setting process [19, 25]. Liu et al. [26] demonstrated that the addition of PVA to a CSC can delay the inflection point (the point before reaching the hydration stage). Additionally, it can exert an inhibitory or fluctuating effect on the pre‐stable period (the period before complete setting). However, its inhibitory effect, which is achieved by surrounding and covering the powder particles, is minimal during the main phase and peak hydration. The long‐chain macromolecular structure of PVA has been reported to accelerate the slowdown phase of cement hydration. In addition, PVA promotes a more homogeneous distribution of cement particles and reduces particle agglomeration, thereby enhancing hydration during this stage. Although PVA is generally regarded as a hydration‐retarding agent, its inhibitory effect appears to be limited and may even decrease the interval between the initial and final setting times [26]. The adhesive properties of PVA, which may contribute to the improvement of cement consistency, are attributed to its hydrophilicity and high molecular weight. In contrast, Noh et al. showed that the addition of PVA to CSC resulted in an increase in setting time. It has been postulated that polymers, such as PVA, which have many hydroxyl groups, are adsorbed onto the surface of the cement particles through hydrogen bonding. These polymers cover the surface of the cement particles, reducing their contact with water and thus reducing the hydration process [19].

No standardized method has been established for assessing the handling properties of these cements. However, cement workability can be evaluated using flowability or spread diameter measurements. The improved workability associated with PVA incorporation has been attributed to both the ball‐bearing and dispersing effects of the polymer [25]. Even at low concentrations, PVA reduces the friction between cement particles, resulting in lower yield stress. In addition, it promotes a more uniform particle distribution and limits flocculation, which may further contribute to the reduction in yield stress. Collectively, these mechanisms improve the rheological behavior of the cement [7]. Determining the optimal polymer concentration is essential. Previous studies have shown that polymer concentrations above the optimum range increase viscosity and consequently reduce cement workability. At higher concentrations, the polymer not only adsorbs on the surface of the cement but also spreads independently within the cement structure. This can lead to an increase in the friction between the cement particles due to the entanglement of polymer molecules between them [25]. The use of the polymers at a concentration of 3 wt% generally increased the flowability of the cement. In order to ascertain the optimum polymer concentration, it is advisable to design another study with higher polymer concentrations. It is also important to monitor the quantity of water used in the liquid component of each group. Allahverdi et al. [25] demonstrated that alterations in the quantity of water in different polymer concentrations can influence the physical and mechanical properties of the cement.

In the present study, the amount of water was selected to achieve optimal handling characteristics of the cement. The polymer concentration was chosen based on previous reports [27–30]. With regard to the impact of polymer addition on cement injectability, it is crucial to consider not only the effect of the polymer on cement viscosity but also the setting time. The enhanced injectability of a polymer‐modified cement may be attributed to the prolongation of the setting time, which is a consequence of polymer incorporation [31, 32]. Given the long setting times observed for the examined cements, it seems that a focus on the viscosity factor and its effect is more relevant than on setting time alone. All groups containing the polymer demonstrated improved injectability, although this was not statistically significant. The results of the flow test indicate that the observed improvements in injectability were likely due to the effect of the polymer on cement viscosity. A reduction in viscosity can enhance flowability and injectability [31]. The addition of all three polymers resulted in a reduction in the compressive strength when compared to the control group. However, no statistically significant difference was observed. When powder and liquid containing polymer are mixed, it is possible for bubbles to be formed in the cement mixture. These bubbles can lead to a decrease in compressive strength [7, 19]. However, it was observed that PVA resulted in a very small decrease in compressive strength, while this decrease was higher for the PVP and PCE groups. The lower amount of water used in the PVA and control groups may be the reason for this finding. Given the type of application of these cements, which are predominantly used as retrograde filling material or liner, the reduction in compressive strength values can be overlooked in favor of obtaining properties such as better workability and a shorter setting time. It is important to note that an increase in compressive strength has also been reported following the use of various polymers, including PVA. The difference between the various methodologies employed, the composition of the powder and liquid, and especially the percentage of polymer used can be significant contributing factors in the observed discrepancies in outcomes [5, 26, 33]. Some researchers have suggested that the observed increase in strength is due to a reduction in porosity and the formation of products resulting from the reaction of PVA with cement that fill the pore spaces. However, others have reported a decrease in strength due to the formation of bubbles during the mixing process [34]. The study by Liu et al. indicates that an increase in the polymer concentration up to 1% of PVA results in an enhancement of the compressive strength of the cement. Nevertheless, an increase in the polymer concentration beyond this threshold results in a reduction in strength. As the polymer concentration rises, it may cover the cement particles, thereby preventing complete hydration [26]. The present study demonstrated that the addition of 1 wt% of polymers resulted in a shorter setting time compared to 3 wt%. However, the obtained results indicated that the addition of 3 wt% polymer improved cement consistency while still maintaining a considerable reduction in setting time without any detrimental effect on the compressive strength.

Assuming that the polymer slows down the cement hydration process, it is logical that the compressive strength of the groups containing the polymer will increase with time and the progress of the setting process. It is better to carry out another study in this field on specimens after 14 and 28 days of mixing. The addition of polymer, due to its high tensile strength and toughness, may have a greater effect on increasing the flexural strength of the material than on the compressive strength [26]. In the present study, the compressive strength was evaluated.

With regard to the impact of PCE on the setting process, a similar contradictory trend is observed in the studies. In a previous study on the addition of PCE to Portland cement liquid, it was demonstrated that by adding different concentrations of polymer up to 2.4 vol% and also reducing the ratio of liquid to powder, the setting time is reduced compared to Portland cement. This issue was attributed to the distinctive structure of this polymer and the mechanism by which it interacts with cement particles. As in the aforementioned study, the present study also demonstrated that lower polymer concentrations led to a greater reduction in setting time [13]. However, Lothenbach [24] et al.’s study found that the addition of PCE had no effect on the hydration process of MTA cement. The results of our study demonstrated that the addition of the PCE polymer had a favorable effect on the setting time and flowability of the material in comparison to the control group. PCE is a superplasticizer. This polymer is a high molecular weight anionic surfactant with a long hydrocarbon chain containing a large number of polar groups. It has an anionic polyacrylic acid backbone that can adsorb onto cement particles, while its polyethylene oxide side chains contribute to the formation of a steric repulsion mechanism. This mechanism promotes repulsion between cement particles, thereby improving particle dispersion and preventing agglomeration [35].

This process facilitates the improvement of flowability [22]. Furthermore, this polymer can interact with calcium cement through carboxylic groups (in methacrylic acid) [35]. By reducing the surface tension of water, PCE can also enhance the flowability, thereby improving the workability of the cement. However, the precise mechanism by which PCE affects cement hydration remains a matter of controversy. Some studies have indicated that PCE may delay or have no effect on cement hydration, while others have observed the opposite. The precise mechanism by which this effect occurs is not yet fully understood [24, 36]. Conversely, it has been proposed that calcium silicate hydrate particles can act as an accelerator, thereby increasing the initial hydration speed of calcium silicate particles. However, the large size of these particles acts as a significant obstacle to achieving this goal.

Polymeric dispersants, such as PCE, have been shown to increase the surface area by reducing the particle agglomeration. Consequently, the seeding effect results in an increase in the hydration of di‐ and tricalcium silicate [37], thereby reducing the setting time. In the present study, none of the polymer‐containing groups showed an improvement in compressive strength compared with the control cement despite the favorable effects observed on handling and flow‐related properties. This finding contrasts with the majority of previous studies [13, 26], which have reported an increase in compressive strength following the incorporation of polymeric dispersants. The incorporation of the PCE polymer has been demonstrated to enhance the strength of cement by reducing the ratio of liquid to powder and the heat generated during hydration. This results in a reduction in the shrinkage of the cement [13]. Nevertheless, the present study did not yield any such a result. It appears that the concentration of the PCE polymer and the liquid‐to‐powder ratio remain critical factors. At higher concentrations, such as those evaluated in the present study, compressive strength decreases, which is attributed to the phenomenon of bleeding and segregation [38]. Moreover, as with PVA, the beneficial effect of this polymer is more closely related to an increase in bending strength than compressive strength [39].

PVP is a high‐molecular‐weight polymer consisting of a carbon‐based backbone with relatively hydrophobic character and pendant hydrophilic pyrrolidone groups. This polymer, like PCE, can act as a dispersant and surfactant in different colloidal systems and can reduce particle agglomeration [40, 41]. It can be concluded that the positive effect of PVP on the setting process and flowability and, as a result, the workability of the material can be justified in a similar way to PCE. It is claimed that PVP prevents the separation of free water from the cement slurry, thereby facilitating the completion of the hydration process [42]. Despite these interpretations, the mechanism of action of this polymer has not yet been determined exactly. In the present study, the most favorable consistency was reported with the cement containing 3 wt% PVP, which was also consistent with the result obtained from the flowability test. Similarly, Jin et al. demonstrated that the addition of PVP to calcium phosphate cement leads to an improvement in the rheological properties of the cement [43]. Although the combination of PVP and PVA with PCE had a less pronounced effect on setting time, it significantly enhanced the flowability compared to the control group. However, the findings of the present study indicated that the use of a single type of polymer may be sufficient to achieve adequate workability. The manner in which PVP decreased the compressive strength is similar to that implied for the other two polymers. Nevertheless, it appears that the percentage of polymer added remains a key factor in this case. It has been found that the addition of less than 1% of PVP is effective in increasing the mechanical properties of cement [44]. It is essential to consider the molecular structure of the polymer when selecting a polymer. Previous studies have demonstrated that the degree of hydrolysis of the PVA polymer, the degree of polymerization, and the molecular weight of the polymer can significantly affect the final properties of the cement [7].

In this study, a highly hydrolyzed PVA polymer was employed. PVA is a hydrophilic polymer with a high molecular weight that is capable of evenly dispersing among the powder particles and connecting them. Consequently, the volume shrinkage of cement is reduced during the hydration and coagulation process of cement. This may contribute to a reduction in the number and size of microcracks and consequently decrease the possibility of water penetration [26]. It can be postulated that this phenomenon may be the reason why, despite the high release of calcium and silicon ions, the integrity of cement‐containing PVA is maintained and less weight loss is observed compared to the control and PVP groups. In all groups, the highest amount of calcium and silicon ion release is observed on the first day, and from the first to the seventh day, the amount of ion release decreases. The increase in pH is mainly associated with the release of calcium and hydroxyl ions (production of calcium hydroxide following the hydration reaction of CSC) [45]. Therefore, a similar trend was observed for pH and calcium‐ion release. The lower pH of the PVA group on the seventh day compared to the other two groups may be due to the specific interaction of this polymer with CSC. It has been proposed that residual acetate groups in PVA may hydrolyze during the hydration process of CSC. The produced acetic acid then reacts with calcium ions, forming Ca(CH_3_COO)_2_. Concurrently, hydroxyl groups may interact with calcium ions present in the hydration products. PVA contains a small amount of residual acetate groups and polyvinyl acetate, which can reduce the pH of the solution and prevent the production of hydration products in the early stages [46]. Therefore, it is also necessary to evaluate ion release and pH values in the early hours following setting.

Zhang et al. [47] reported that a calcium silicate/calcium phosphate/PVA composite cement maintained its structural integrity despite exhibiting a degradation rate of 49% over a 56‐day period. The release of calcium and silicon ions has been associated with cellular differentiation and extracellular matrix remineralization. Owing to the presence of numerous hydroxyl groups in its structure, PVA may promote the formation of strong intermolecular interactions when incorporated at appropriate concentrations, thereby reducing cement brittleness. In this regard, PVA may function as an effective binding agent within the cement matrix [47]. These findings are consistent with the data obtained in the present study. Despite the higher solubility of the distilled water and PVP groups, particularly on the first day, the amount of ion release in these groups was significantly lower than in the PVA group. This is consistent with the unique characteristics of the PVA polymer, as previously discussed. The XRD patterns indicated that the addition of the polymer did not alter crystallinity. It is notable that the products resulting from interactions between the polymer and the powder or the hydration of calcium silicate may exhibit a noncrystalline nature that cannot be detected in XRD [19]. Hydrophilic polymers may either interact with powder particles and the products of hydration or simply physically coat the particles as a thin film. The specific nature of the interactions that occur is influenced by a number of variables, including the type of the polymer and its quantity. The chemical mechanisms involved remain poorly understood due to the amorphous nature of the reaction product. It is conceivable that the formed calcium compounds may occupy the cement porosity spaces [34, 48]. The SEM/EDS analysis of the set cements from the distilled water group demonstrated the formation of hydrated calcium silicate. One day after immersion in SBF, globular deposits of calcium phosphate were observed in all groups. These precipitates were also observed on the seventh day and were confirmed by EDX analysis. Since calcium phosphate deposits are amorphous or poorly crystalline, they are not detected in XRD. In all groups, cubic or polygonal precipitates, as well as spherulites, were observed on the first and seventh days. These precipitates were related to calcium carbonate. The amount of spherulites was gradually increased until the seventh day, as confirmed by observing the peak of calcium carbonate in XRD analysis. This peak was more intense on the seventh day. The process of intensification of this peak was found to be faster for the PVA group. The formation of calcium carbonate through the reaction of calcium‐containing phases with environmental carbon dioxide has also been confirmed by previous studies [49, 50]. If the cement was immersed for more days, it is possible to observe the formation of hydroxyapatite and hydroxyapatite carbonate peaks in the XRD graphs.

The formation of calcium carbonate has been suggested to facilitate or precede the formation of hydroxyapatite [49]. The formation of this material along with calcium phosphate precipitations is indicative of the biomimetic behavior of the cements. It is noteworthy that in bones and teeth, along with hydroxyapatite, the calcium carbonate is observed [51]. It is noteworthy that the absence of a calcium hydroxide peak in the XRD analysis of the present study has also been observed in some commercial products. The formation of this phase depends on a variety of variables, including the chemical composition of the cement and the dimensions and morphology of the particles. It can also be the consequence of an incomplete hydration process or sub‐saturation concentrations of calcium hydroxide, which cannot be detected [52, 53].

## 5. Conclusion

1. The observed results showed that by adding each of these polymers, especially when added alone, the consistency of the obtained cement was improved.
2. The observed results showed that by adding each of these polymers alone or in combination with PCE, the setting time was reduced.
3. Although the addition of polymers reduced the compressive strength, this reduction was not significant.
4. Although the addition of selected polymers (PVA 3 wt%, PVP 3 wt%, PCE 1 wt% + PVA 3 wt%, and PCE 1 wt% + PVP 3 wt%) improved flowability, the effect on injectability was not significant.
5. Overall, the addition of PVA 3 wt% or PVP 3 wt% polymers separately in the liquid component of CSC improved the properties of this cement.
6. The addition of PVA 3 wt% or PVP 3 wt% polymers did not interfere with the release of calcium ions or the formation of calcium phosphate precipitates after immersion in SBF.

## Author Contributions

**Farhood Najafi:** conceptualization, methodology, supervision. **Soolmaz Heidari:** conceptualization, methodology, writing – review and editing. **Nazanin Zargar:** writing – original draft, writing – review and editing. **Mojgan Feli:** investigation, writing – original draft, writing – review and editing. **Shokoufeh Borhan:** investigation. **Saeede Zadsirjan:** writing – original draft, writing – review and editing. **Negar Parvane Dehkordi:** investigation. **Golnaz Tayebi:** investigation.

## Funding

This research did not receive any specific grant from funding agencies in the public, commercial, or not‐for‐profit sectors.

## Disclosure

All authors have read and approved the final version of the manuscript. Mojgan Feli, the corresponding author, had full access to all of the data in this study and takes complete responsibility for the integrity of the data and the accuracy of the data analysis.

## Ethics Statement

This study was an in vitro laboratory investigation and did not involve human participants, human tissues, patient data, or animals. Therefore, ethical approval and informed consent were not required.

## Conflicts of Interest

The authors declare no conflicts of interest.

## Data Availability

The data used to support the findings of this study were supplied by corresponding author under license, and data will be available on request. Requests for access to these data should be made to corresponding author.
